# Supplementary material for: Coordination of bacterial cell wall and outer membrane biosynthesis
Source: Nature. 2023 Mar 1;615(7951):300–4. doi: 10.1038/s41586-023-05750-0 (PMC9995270; doi:10.1038/s41586-023-05750-0)
Supplement: Supplementary file 1 — This document contains Supplementary Methods, Tables 1–5, and Supplementary References. [file 41586_2023_5750_MOESM1_ESM.pdf]

nature portfolio

https://doi.org/10.1038/s41586-023-05750-0

Supplementary information

Coordination of bacterial cell wall and outer membrane biosynthesis

In the format provided by the authors and unedited

SUPPLEMENTARY METHODS

Plasmid construction

pKH3 [ΩΔ*ftsH sacB gent*] – The upstream and downstream flanking regions of the *ftsH* locus were amplified from PAO1 gDNA using primers K5/K6 and K7/K8. The resulting fragments were ligated using Gibson assembly and subsequently inserted into the EcoRI/XbaI sites of pEXG2.

pKH4 [Ω*6-his-lpxC sacB gent*] – Upstream and downstream regions flanking the 5' end of *lpxC* were amplified from PAO1 gDNA using primers K9/K10 and K11/K12. Primer K10 encoded the introduced 6-his tag. The resulting fragments were ligated using Gibson assembly and subsequently inserted into the EcoRI/XbaI sites of pEXG2.

pKH19 [P_{ara}-*6-his*^{Pa}*lpxC amp*] – The ^{Pa}*lpxC* locus was amplified from *P. aeruginosa* PAO1 gDNA using primers K27/K15 and subsequently ligated into the EcoRI/HindIII sites of pHerd20T.

pKH20 [P_{ara}-*6-his*^{Ec}*lpxC amp*] – The ^{Ec}*lpxC* locus was amplified from *E. coli* MG1655 gDNA using primers K24/K25 and subsequently ligated into the EcoRI/HindIII sites of pHerd20T.

pKH23 [P_{ara}-^{Pa}*lpxC amp*] – The ^{Pa}*lpxC* locus was amplified from PAO1 gDNA using primers K54/K55 and subsequently ligated into the KpnI/SphI sites of pHerd20T.

pKH24 [P_{ara}-^{Ec}*lpxC amp*] – The ^{Ec}*lpxC* locus was excised from the XbaI/HindIII sites of pPR111 and subsequently ligated into the XbaI/HindIII sites of pHerd20T.

pKH37 [P_{lac}-^{Pa}*murA(WT) gent*] – The ^{Pa}*murA* open reading frame along with the 17 base pairs immediately upstream was synthesized by Genscript and inserted into the SacI and XbaI sites of pLSM11.

pKH38 [P_{lac}-^{Pa}*murA(C117S) gent*] – pKH37 was used as a template for site directed mutagenesis performed by Genscript. Specifically, the cysteine at codon 117 (TGC in WT) was mutated to a serine (AGC).

pKH39 [ΩΔ*PA4701 sacB gent*] – The upstream and downstream flanking regions of the *PA4701* locus were amplified from PAO1 gDNA using primers K78/79 and K80/K81. The resulting fragments were ligated using Gibson assembly and subsequently inserted into the HindIII/BamHI sites of pEXG2.

pKH58 [P_{ara}-^{Pa}*murA amp*] – The ^{Pa}*murA* locus was amplified from PAO1 gDNA using primers K98/K112 and subsequently ligated into the XmaI/SphI sites of pHerd20T.

pKH69 [P_{lac}-*FLAG*-^{Pa}*murA(WT) amp*] – The ^{Pa}*murA* locus was amplified from PAO1 gDNA using primers K148/K149 and subsequently ligated into the XmaI/HindIII sites of pPSV38.

pKH86 [ΩΔ*murA sacB gent*] – The upstream and downstream flanking regions of the *murA* locus were amplified from PAO1 gDNA using primers K166/K167 and K168/K169. The resulting fragments were ligated using Gibson assembly and subsequently inserted into the EcoRI/XbaI sites of pEXG2.

pKH87 [ΩΔ*murB sacB gent*] – The upstream and downstream flanking regions of the *murB* locus were amplified from PAO1 gDNA using primers K170/K171 and K172/K173. The resulting fragments were ligated using Gibson assembly and subsequently inserted into the EcoRI/XbaI sites of pEXG2.

pKH88 [*Tn7::*$P_{toplacuv5}$-*murA gent*] – The ^{Pa}*murA* locus was amplified from PAO1 gDNA using primers K174/K112 and subsequently ligated into the BamHI/SphI sites of pKHT105.

pKH90 [P_{T7}-*his-SUMO-FLAG*-^{Pa}*lpxC amp*] – The ^{Pa}*lpxC* locus was amplified from PAO1 gDNA using primers K179/K180. pAM205, encoding His-SUMO-FLAG-^{Taq}*rodA* was linearized by amplification with primers K177/K178 so as to exclude the ^{Taq}*rodA* open reading frame. The two resulting fragments were ligated using Gibson assembly.

pKH97 [P_{T7}-*his*-^{Pa}*murA kan*] – The ^{Pa}*murA* locus was amplified from PAO1 gDNA using primers K193/K194. pET28a was linearized by amplification with primers K183/K184 and the two resulting fragments were ligated using Gibson assembly.

pKH100 [*P_{lac}-FLAG-^{Pa}murA(C117S) gent*] – The upstream and downstream portions of ^{Pa}*murA* were amplified from PAO1 gDNA using primers K148/K108 and K109/K149. K108 and K109 encoded the C117S substitution. The resulting fragments were ligated using Gibson assembly and subsequently ligated into the XmaI/HindIII sites of pPSV38.

pKH103 [*P_{T7}-his-^{Pa}murA(C117S) kan*] – The upstream and downstream portions of ^{Pa}*murA* were amplified from PAO1 gDNA using primers K193/K108 and K109/K194. K108 and K109 encoded the C117S substitution. pET28a was linearized by amplification with primers K183/K184 and the three resulting fragments were ligated using Gibson assembly.

pKH155 [*Tn7::$P_{toplacdn1}$-murB gent*] – The ^{Pa}*murB* locus was amplified from PAO1 gDNA using primers K175/K176 and subsequently ligated into the BamHI/SphI sites of pKHT104.

pKH156 [*P_{T7}-his-SUMO-FLAG-^{Ec}lpxC amp*] – The ^{Ec}*lpxC* locus was amplified from *E. coli* MG1655 gDNA using primers K202/K203. pAM205, encoding His-SUMO-FLAG-^{Taq}*rodA* was linearized by amplification with primers K177/K178 so as to exclude the ^{Taq}*rodA* open reading frame. The two resulting fragments were ligated using Gibson assembly.

pKH157 [*P_{T7}-his-^{Ec}murA kan*] – The ^{Ec}*murA* locus was amplified from *E. coli* MG1655 gDNA using primers K204/K205. pET28a was linearized by amplification with primers K183/K184 and the two resulting fragments were ligated using Gibson assembly.

pKH161 [*Ω^{Pa}murA(C117S) sacB gent*] – Upstream and downstream regions flanking ^{Pa}*murA* residue 117 were amplified from PAO1 gDNA using primers K166/K108 and K109/K218. Primers K108/K109 encoded the C117S substitution. The resulting fragments were ligated using Gibson assembly and subsequently inserted into the EcoRI/BamHI sites of pEXG2.

pKH189 [*P_{T7}-his-SUMO-FLAG-^{Aba}lpxC amp*] – The ^{Aba}*lpxC* locus was amplified from *A. baumannii* ATCC 17978 gDNA using primers K223/K224. pAM205, encoding His-SUMO-FLAG-^{Taq}*rodA* was linearized by amplification with primers K177/K178 so as to exclude the ^{Taq}*rodA* open reading frame. The two resulting fragments were ligated using Gibson assembly.

pKH194 [*P_{T7}-his-^{Aba}murA kan*] – The ^{Aba}*murA* locus was amplified from *A. baumannii* ATCC 17978 gDNA using primers K225/K226. pET28a was linearized by amplification with primers K183/K184 and the two resulting fragments were ligated using Gibson assembly. A stop codon was subsequently introduced into the AbamurA open reading frame using the QuikChange lightning kit (Agilent 210514) with primers K238/K239.

pKH215 [*P_{T7}-his-^{Pa}murA(K22R) kan*] – The *His-^{Pa}murA* open reading frame in pKH97 was mutagenized using the QuikChange lightning kit (Agilent 210514) with primers K284/K285.

pKH216 [*P_{T7}-his-^{Pa}murA(C117R) kan*] – The *His-^{Pa}murA* open reading frame in pKH97 was mutagenized using the QuikChange lightning kit (Agilent 210514) with primers K286/K287.

pKH217 [*P_{T7}-his-^{Pa}murA(I119F) kan*] – The *His-^{Pa}murA* open reading frame in pKH97 was mutagenized using the QuikChange lightning kit (Agilent 210514) with primers K288/K289.

pKH218 [*P_{T7}-his-^{Pa}murA(D308N) kan*] – The *His-^{Pa}murA* open reading frame in pKH97 was mutagenized using the QuikChange lightning kit (Agilent 210514) with primers K290/K291.

pKH219 [*P_{T7}-his-^{Pa}murA(Q310R) kan*] – The *His-^{Pa}murA* open reading frame in pKH97 was mutagenized using the QuikChange lightning kit (Agilent 210514) with primers K292/K293.

pKH220 [*P_{T7}-his-^{Pa}murA(R334C) kan*] – The *His-^{Pa}murA* open reading frame in pKH97 was mutagenized using the QuikChange lightning kit (Agilent 210514) with primers K294/K295.

pKH221 [*P_{T7}-his-^{Pa}murA(R334S) kan*] – The *His-^{Pa}murA* open reading frame in pKH97 was mutagenized using the QuikChange lightning kit (Agilent 210514) with primers K296/K297.

pKH247 [*P_{T7}-his-^{Pa}murA(G58D) kan*] – The *His-^{Pa}murA* open reading frame in pKH97 was mutagenized using the QuikChange lightning kit (Agilent 210514) with primers K379/K380.

pKH248 [*P_{T7}-his-^{Pa}murA(E406K) kan*] – The *His-^{Pa}murA* open reading frame in pKH97 was mutagenized using the QuikChange lightning kit (Agilent 210514) with primers K381/K382.

pKH251 [*P_{lac}-^{Pa}murA(C117S,G58D) gent*] – The ^{Pa}*murA(C117S)* open reading frame in pKH38 was mutagenized using the QuikChange lightning kit (Agilent 210514) with primers K379/K380.

pKH252 [P_{lac}-^{Pa}*murA(C117S,E406K) gent*] – The ^{Pa}*murA(C117S)* open reading frame in pKH38 was mutagenized using the QuikChange lightning kit (Agilent 210514) with primers K381/K382.

pKH257 [P_{lac}-*FLAG*-^{Pa}*murA(C117S,G58D) gent*] – The *FLAG*-^{Pa}*murA(C117S)* open reading frame in pKH100 was mutagenized using the QuikChange lightning kit (Agilent 210514) with primers K379/K380.

pKH258 [P_{lac}- *FLAG*-^{Pa}*murA(C117S,E406K) gent*] – The *FLAG*-^{Pa}*murA(C117S)* open reading frame in pKH100 was mutagenized using the QuikChange lightning kit (Agilent 210514) with primers K381/K382.

pKH261 [P_{T7}-*his-SUMO-FLAG*-^{Mku}*lpxC amp*] –A DNA fragment containing the *Magnetospirllum kuznetsovii lpxC* open reading frame codon optimized for expression in *E. coli* and flanked by regions homologous to primers K177 and K178 was synthesized as a gBlock by IDT. The resulting fragment was ligated into pKH90 linearized by PCR with primers K177 and K178. The gBlock sequence can be found in table S5.

pKH262 [P_{T7}-*his-SUMO-FLAG*-^{Xba}*lpxC amp*] –A DNA fragment containing the *Magnetospirllum kuznetsovii* (TaxID 2053833) *lpxC* open reading frame codon optimized for expression in *E. coli* and flanked by regions homologous to primers K177 and K178 was synthesized as a gBlock by IDT. The resulting fragment was ligated into pKH90 linearized by PCR with primers K177 and K178. The gBlock sequence can be found in table S5.

pKH264 [P_{T7}-*his*-^{Mku}*murA kan*] –A DNA fragment containing the *Magnetospirllum kuznetsovii* (TaxID 2053833) *murA* open reading frame codon optimized for expression in *E. coli,* driven by an optimized ribosome binding site, and flanked by regions homologous to primers K183 and K184 was synthesized as a gBlock by IDT. The resulting fragment was ligated into pKH97 linearized by PCR with primers K183 and K184. The gBlock sequence can be found in table S5.

pKH265 [P_{T7}-*his*-^{Xba}*murA kan*] –A DNA fragment containing the *Xanthomonadales bacterium* (TaxID 2006849) *murA* open reading frame codon optimized for expression in *E. coli,* driven by an optimized ribosome binding site, and flanked by regions homologous to primers K183 and K184 was synthesized as a gBlock by IDT. The resulting fragment was ligated into pKH97 linearized by PCR with primers K183 and K184. The gBlock sequence can be found in table S5.

pKH267 [P_{T7}-*his-SUMO-FLAG*-^{Lpn}*lpxC amp*] –A DNA fragment containing the *Legionella pneumophila subsp. Pneumophila* ATCC 33152 *lpxC* open reading frame codon optimized for expression in *E. coli* and flanked by regions homologous to primers K177 and K178 was synthesized as a gBlock by IDT. The resulting fragment was ligated into pKH90 linearized by PCR with primers K177 and K178. The gBlock sequence can be found in table S5.

pKH268 [P_{T7}-*his*-^{Lpn}*murA kan*] – A DNA fragment containing the *Legionella pneumophila subsp. Pneumophila* ATCC 33152 *murA* open reading frame codon optimized for expression in *E. coli,* driven by an optimized ribosome binding site, and flanked by regions homologous to primers K183 and K184 was synthesized as a gBlock by IDT. The resulting fragment was ligated into pKH97 linearized by PCR with primers K183 and K184. The gBlock sequence can be found in table S5.

pKH269 [P_{ara}-^{Pa}*murA(G58D) amp*] – The ^{Pa}*murA* open reading frame in pKH58 was mutagenized using the QuikChange lightning kit (Agilent 210514) with primers K379/K380.

Strain construction

***P. aeruginosa* transformation** – 1 mL of *P. aeruginosa* cultures grown overnight at either 30°C or 37°C were pelleted by centrifugation at 9,391 x g and washed 4 times in an equal volume of 300 mM sucrose. Cells were resuspended in 50 µL of 300 mM sucrose, mixed with approximately 50 ng of DNA, and subsequently electroporated in 2 mm cuvettes (Genesee Scientific 40-101) at 2.5 kV using a Bio-Rad MicroPulser. Unless stated otherwise, cells were recovered in 1 mL LB rotating end-over-end at 30°C for 1 hr and transformants were selected by plating the cells onto LB agar supplemented with the appropriate antibiotic.

Allelic replacement – *E. coli* SM10 strains harboring pEXG2 derivatives were used as donors in conjugations in which *P. aeruginosa* was a recipient. Transconjugants were selected by growth on Vogel-Bonner minimal medium (3 g trisodium citrate, 2 g citric acid, 10 g K_2HPO_4, 3.5 g $NaNH_4PO_4{\bullet}4H_2O$, and 15 g agar per 1 L) supplemented with 30 µg/mL gentamycin. The resulting transconjugants were allowed to excise the plasmid by growth in LB at 37°C and *sacB*$^-$ isolates were obtained by selection on LB agar plates supplemented with 5% (w/v) sucrose. The presence of the desired mutation was then validated as described below.

***6-his-^{Pa}lpxC* –** pKH4 was introduced into *P. aeruginosa* PAO1 by conjugation and excised as described above to create PA1018. The presence of the *6-his-^{Pa}lpxC* mutation was verified by PCR amplification of the *lpxC* locus with primers K9/K12 and subsequent sanger sequencing with primer K17. pKH3 was introduced into PA1018 and excised as described above to create PA1037. The presence of Δ*ftsH* was verified by PCR with primers K5/K21.

Δ*PA4701* – pKH39 was introduced into *P. aeruginosa* PAO1 by conjugation and excised as described above to create PA4701. The presence of Δ*PA4701* was verified by PCR with primers K81/K100.

Δ^{Pa}*murA* – pKH88 was co-transformed with pTNS2 into *P. aeruginosa* PAO1. Gentamycin-resistant colonies were selected and integration into attTn7 was confirmed by PCR with primers 67/68. pFlp2 was introduced into the resulting strain and the *gent*^{R} cassette was excised, resulting in PA1117. pKH86 was introduced into PA1117 by conjugation and excised in the presence of 1 mM IPTG as described above to create PA1118. The presence of Δ^{Pa}*murA* was verified by PCR with primers K187/K169. PA1118 was propagated in the presence of 1 mM IPTG.

Δ^{Pa}*murB* – pKH155 was co-transformed with pTNS2 into *P. aeruginosa* PAO1. Gentamycin-resistant colonies were selected and integration into attTn7 was confirmed by PCR with primers 67/68. pFlp2 was introduced into the resulting strain, the *gent*^{R} cassette was excised, and pKH87 was introduced by conjugation and subsequently excised in the presence of 1 mM IPTG as described above to create PA1135. The presence of Δ^{Pa}*murB* was verified by PCR with primers K188/K173. PA1135 was propagated in the presence of 1 mM IPTG.

^{Pa}murA(C117S) – pKH161 was introduced by conjugation into PA1117 and subsequently excised in the presence of 1 mM IPTG as described above to create PA1162. The presence of ^{Pa}*murA(C117S)* at the native site was verified by amplification of the *murA* locus with primers K187/K106 and subsequent sanger sequencing with primer K187. We found that the ^{Pa}*murA(C117S)* allele was not stably maintained in LB at 37°C and reverted to the ^{Pa}*murA(WT)* allele with high frequency under those conditions. To mitigate this instability, PA1162 and its derivatives were propagated at 30°C in M9 media[1] supplemented with 1 mM IPTG.

Protein Purification

His-MurA – 10 mL of an overnight culture of *E. coli* strain ER2566 harboring pKH97, pKH103, pKH157, pKH194, pKH264, pKH265, or pKH268 was subcultured into 1 L Terrific broth (24 g yeast extract, 12 g tryptone, 0.4% glycerol, 2.31 g KH_2PO_4 and 12.5 g K_2HPO_4 per 1 L) and allowed to grow at 37°C until reaching an OD_{600} of 0.5-0.6. Expression of 6-his-MurA was then induced by the addition of IPTG to 1 mM and the culture was grown at 20°C for approximately 20 hours. Cells were pelleted by centrifugation at 12,000 xg for 10 min, the pellet was resuspended in 45 mL Lysis buffer A (25 mM Tris pH 8.0, 100 mM NaCl, 5 mM imidazole) and cells were subsequently lysed by 2 passages through a continuous flow cell disruptor (Constant Solutions, Ltd) at 25,000 psi. The extract was clarified by two successive centrifugations at 32,000 rpm for 20 min at 4°C using a 45 Ti rotor (Beckman Coulter) and subsequently bound to Ni-NTA agarose resin (Qiagen 30230). The resin was washed with 10 column volumes of wash buffer A (25 mM Tris pH 8.0, 100 mM NaCl, 25 mM imidazole) and protein was eluted in four 5 mL fractions of lysis buffer containing increasing concentrations of imidazole: 50 mM, 100 mM, 250 mM, and 500 mM. One fraction containing the highest concentration of 6-his-MurA as assayed by SDS-PAGE and subsequent Coomassie staining was dialyzed overnight at 4°C in 1L MurA storage buffer A (25

mM Tris pH 8.0, 150 mM NaCl, 0.5 mM DTT, 10% glycerol) using SnakeSkin dialysis tubing (Thermo 68700). Dialyzed products were flash frozen in liquid nitrogen and stored at -80°C. The concentration of 6-His-MurA orthologs were determined using Quick Start Bradford 1X Dye Reagent (Bio-Rad 5000205) and a standard curve of BSA. Purified proteins were resolved on by SDS-PAGE on a 15% polyacrylamide and protein was detected with Coomassie staining (**Extended Data 5a**)

FLAG-LpxC – 10 mL of an overnight culture of *E. coli* strain ER2566 harboring pAM174 as well as pKH90, pKH156, pKH189, pKH261, pKH262, or pKH267 was subcultured into 1 L Terrific broth (24 g yeast extract, 12 g tryptone, 0.4% glycerol, 2.31 g KH_2PO_4 and 12.5 g K_2HPO_4 per 1 L) and allowed to grow at 37°C until reaching an OD_{600} of 0.5-0.6. Expression of FLAG-LpxC and Ulp1 were simultaneously induced by the addition of 1 mM IPTG and 0.05% arabinose, and the culture was grown at 30°C for an additional 4 hours. Cells were pelleted by centrifugation at 12,000 xg for 10 min, the pellet was resuspended in 45 mL Lysis buffer B (20 mM HEPES pH 7.5, 500 mM NaCl, 20 mM $MgCl_2$, 0.5 mM DTT) and cells were subsequently lysed by 2 passages through a continuous flow cell disruptor (Constant Solutions, Ltd) at 25,000 psi. The extract was clarified by two successive centrifugations at 32,000 rpm for 20 min at 4°C using a 45 Ti rotor (Beckman Coulter), $CaCl_2$ was added to 2 mM, and supernatant was bound to a homemade M1 α-Flag antibody resin. The resin was washed with 25 column volumes of wash buffer B (20 mM HEPES pH 7.5, 500 mM NaCl, 2 mM $CaCl_2$) and protein was eluted with 5 column volumes of elution buffer B (20 mM HEPES pH 7.0, 500 mM NaCl, 5 mM EDTA pH 8.0, 100 µg/mL FLAG peptide). The elution was concentrated by centrifugation at 4°C using 10 kDa molecular weight cutoff centrifugal filters (Amicon UFC801024). The concentrated protein was next subjected to size exclusion chromatography using an ActaPure system equipped with a Superdex™ 75 10/300 GL column equilibrated with SEC buffer (20 mM HEPES, 500 mM NaCl). LpxC peak fractions were pooled and dialyzed overnight at 4°C in 1L LpxC storage buffer (25 mM Tris pH 8.0, 150 mM NaCl, 2 mM DTT, 10% glycerol) using SnakeSkin dialysis tubing (Thermo 68700). Dialyzed products were flash frozen in liquid nitrogen and stored at -80°C. The concentration of Flag-LpxC orthologs were determined using Quick Start Bradford 1X Dye Reagent (Bio-Rad 5000205) and a standard curve of BSA. Purified proteins were resolved on by SDS-PAGE on a 15% polyacrylamide and protein was detected with Coomassie staining (**Extended Data 5a**)

Supplementary Table 1. Proteins that co-purify with [Pa]LpxC identified by mass spectrometry.

Gene Symbol	PAO1 (WT)						PA1018 (6-his-[Pa]LpxC)						PA1009 (6-his-[Ec]LpxC)					
	150 mM NaCl			300 mM NaCl			150 mM NaCl			300 mM NaCl			150 mM NaCl			300 mM NaCl		
	Unique[a]	Total[b]	Sum Intensity[c]	Unique	Total	Sum Intensity	Unique	Total	Sum Intensity	Unique	Total	Sum Intensity	Unique	Total	Sum Intensity	Unique	Total	Sum Intensity
lpxC	6	10	3.8E+06	7	11	3.7E+06	28	657	9.9E+09	29	760	2.8E+10	9	33	3.4E+08	10	33	6.8E+08
murA	2	2	2.6E+05	1	1	1.9E+04	11	17	5.8E+06	21	42	1.2E+08	2	5	9.0E+05	2	2	2.8E+05
PA4701	0	0	NF	0	0	NF	19	14	5.0E+06	19	28	2.4E+07	0	0	NF	0	0	NF

[a]Unique indicates the number of unique peptides identified.
[b]Total indicates the total number of peptides identified.
[c]NF = Not Found

Supplementary Table 2 – Plasmids used in this study

Plasmid	Genotype	Reference or source
pAM174	P_{ara}-6-his-Ulp1 cat	[2]
pAM205	P_{T7}-his-sumo-flag-^{Taq}rodA amp	Kruse Lab, Boston, MA
pET28a	P_{T7} kan	Novagen
pEXG2	sacB gent	[3]
pFLP2	Flp recombinase amp	[4]
pHerd20T	P_{ara} amp	[5]
pKHT104	Tn7::$P_{toplacdn1}$ gent	Dove Lab, Cambridge, MA
pKHT105	Tn7::$P_{toplacuv5}$ gent	Dove Lab, Cambridge, MA
pLSM11	P_{lac}-ftsL gent	[6]
pPR111	P_{lac}-^{Ec}lpxC cat	[7]
pPSV38	P_{lac} gent	[8]
pTNS2	tnsABC+D amp	[9]
pKH3	ΩΔftsH sacB gent	
pKH4	Ω6-his-^{Pa}lpxC sacB gent	
pKH19	P_{ara}-6-his-^{Pa}lpxC amp	
pKH20	P_{ara}-6-his-^{Ec}lpxC amp	
pKH23	P_{ara}-^{Pa}lpxC amp	
pKH24	P_{ara}-^{Ec}lpxC amp	
pKH37	P_{lac}-^{Pa}murA gent	Genscript
pKH37-2A	P_{lac}-^{Pa}murA(K22R) gent	
pKH37-4C	P_{lac}-^{Pa}murA(D49V) gent	
pKH37-1A	P_{lac}-^{Pa}murA(G116S) gent	
pKH37-5R	P_{lac}-^{Pa}murA(C117R) *silent mutation codon 298 CGT>CGC gent	
pKH37-5U	P_{lac}-^{Pa}murA(C117S) gent	
pKH37-3D	P_{lac}-^{Pa}murA(I119F) gent	
pKH37-1B	P_{lac}-^{Pa}murA(L126P) gent	
pKH37-1C	P_{lac}-^{Pa}murA(V166A) *silent mutation codon 283 ATC>ATT gent	
pKH37-1E	P_{lac}-^{Pa}murA(E193G) gent	
pKH37-2B	P_{lac}-^{Pa}murA(D234A) gent	
pKH37-4N	P_{lac}-^{Pa}murA(I236T) gent	
pKH37-4L	P_{lac}-^{Pa}murA(T240I) gent	
pKH37-5D	P_{lac}-^{Pa}murA(D308G) gent	
pKH37-1D	P_{lac}-^{Pa}murA(D308N) *silent mutation codon 218 ATC>ATT gent	
pKH37-5N	P_{lac}-^{Pa}murA(Q310R) gent	
pKH37-5L	P_{lac}-^{Pa}murA(E328G) gent	
pKH37-2J	P_{lac}-^{Pa}murA(V330A) gent	
pKH37-2H	P_{lac}-^{Pa}murA(F331L) gent	
pKH37-4J	P_{lac}-^{Pa}murA(R334C) gent	
pKH37-1L	P_{lac}-^{Pa}murA(R334S) gent	
pKH37-3G	P_{lac}-^{Pa}murA(D372G) *silent mutation codon 83 GTC>GTT gent	
pKH37-3C	P_{lac}-^{Pa}murAA(375T) gent	
pKH37-4I	P_{lac}-^{Pa}murA(L379P) gent	
pKH38	P_{lac}-^{Pa}murA(C117S) gent	Genscript
pKH39	ΩΔPA4701 sacB gent	
pKH58	P_{ara}-murA amp	
pKH69	P_{lac}-FLAG-^{Pa}murA gent	
pKH86	ΩΔmurA sacB gent	
pKH87	ΩΔmurB sacB gent	
pKH88	Tn7::$P_{toplacuv5}$-murA gent	
pKH90	P_{T7}-6-his-SUMO-FLAG-^{Pa}lpxC amp	
pKH97	P_{T7}-6-his-^{Pa}murA kan	
pKH100	P_{lac}-FLAG-^{Pa}murA(C117S) gent	
pKH103	P_{T7}-6-his-^{Pa}murA(C117S) kan	
pKH155	Tn7::$P_{toplacdn1}$-murB gent	

pKH156	*P_{T7}-6-his-SUMO-FLAG-^{Ec}lpxC amp*	
pKH157	*P_{T7}-6-his-^{Ec}murA kan*	
pKH161	*Ω^{Pa}murA(C117S) sacB gent*	
pKH189	*P_{T7}-6-his-SUMO-FLAG-^{Aba}lpxC amp*	
pKH194	*P_{T7}-6-his-^{Aba}murA kan*	
pKH215	*P_{T7}-6-his-^{Pa}murA(K22R) kan*	
pKH216	*P_{T7}-6-his-^{Pa}murA(C117S) kan*	
pKH217	*P_{T7}-6-his-^{Pa}murA(I119F) kan*	
pKH218	*P_{T7}-6-his-^{Pa}murA(D308N) kan*	
pKH219	*P_{T7}-6-his-^{Pa}murA(Q310R) kan*	
pKH220	*P_{T7}-6-his-^{Pa}murA(R334C) kan*	
pKH221	*P_{T7}-6-his-^{Pa}murA(R334S) kan*	
pKH247	*P_{T7}-6-his-^{Pa}murA(G58D) kan*	
pKH248	*P_{T7}-6-his-^{Pa}murA(E406K) kan*	
pKH251	*P_{lac}-^{Pa}murA(C117S,G58D) gent*	
pKH252	*P_{lac}-^{Pa}murA(C117S,E406K) gent*	
pKH257	*P_{lac}-FLAG-^{Pa}murA(C117S,G58D) gent*	
pKH258	*P_{lac}-FLAG-^{Pa}murA(C117S,E406K) gent*	
pKH261	*P_{T7}-6-his-SUMO-FLAG-^{Mku}lpxC amp*	
pKH262	*P_{T7}-6-his-SUMO-FLAG-^{Xba}lpxC amp*	
pKH264	*P_{T7}-6-his-^{Mku}murA kan*	
pKH265	*P_{T7}-6-his-^{Xba}murA kan*	
pKH267	*P_{T7}-6-his-SUMO-FLAG-^{Lpn}lpxC amp*	
pKH268	*P_{T7}-6-his-^{Lpn}murA kan*	
pKH269	*P_{ara}-^{Pa}murA^{G58D} amp*	

Supplementary Table 3 - Strains used in this study

Strain name	Strain #	Genotype[a,b]	Reference or Source
E. coli strains			
ER2566	-	lacZ::T7 gene1 fhuA2 lon ompT gal sulA11 mcr endA1 dcm	New England Biolabs
XL1-red	-	endA1 gyrA96 thi-1 hsdR17 supE44 relA1 lac mutD5 mutS mutT Tn10 (Tetr)a	[10]
P. aeruginosa strains			
PAO1	-	*P aeruginosa* Wild type	Lory Lab, Boston, MA
PAO1/pPSV38	PA239	*[P_{lac}-empty gent]*	
PAO1/pKH19	PA1008	*[P_{ara}-6-his-^{Pa}lpxC amp]*	
PAO1/pKH20	PA1009	*[P_{ara}-6-his-^{Ec}lpxC amp]*	
PAO1/pHerd20T	PA1010	*[P_{ara}-empty amp]*	
PA1018/pPSV38	PA1013	*6-his-^{Pa}lpxC [P_{lac}-empty gent]*	
PA1018	PA1018	*6-his-^{Pa}lpxC*	
PAO1/pKH23	PA1020	*[P_{ara}-^{Pa}lpxC amp]*	
PAO1/pKH24	PA1021	*[P_{ara}-^{Ec}lpxC amp]*	
PAO1/pKH37	PA1035	*[P_{lac}-^{Pa}murA gent]*	
PAO1/pKH38	PA1036	*[P_{lac}-^{Pa}murA(C117S) gent]*	
PA1037	PA1037	*6-his-^{Pa}lpxC ΔftsH*	
PAO1/pKH69	PA1068	*[P_{lac}-FLAG-^{Pa}murA gent]*	
PA1018/pKH69	PA1071	*6-his-^{Pa}lpxC [P_{lac}-FLAG-^{Pa}murA gent]*	
PA1080	PA1080	*ΔPA4701*	
PA1117	PA1117	*attTn7::$P_{TOPLACUV5}$-^{Pa}murA*	
PA1118	PA1118	*Δ^{Pa}murA attTn7::$P_{TOPLACUV5}$-^{Pa}murA*	
PA1018/pKH100	PA1121	*6-his-^{Pa}lpxC [P_{lac}-FLAG-^{Pa}murA(C117S) gent]*	
PA1135	PA1135	*ΔmurB attTn7::$P_{TOPLACDN1}$-murB*	
PAO1/pKH37-2A	PA1136	*[P_{lac}-^{Pa}murA(K22R) gent]*	
PAO1/pKH37-4C	PA1137	*[P_{lac}-^{Pa}murA(D49V) gent]*	
PAO1/pKH37-1A	PA1138	*[P_{lac}-^{Pa}murA(G116S) gent]*	
PAO1/pKH37-5R	PA1139†	*[P_{lac}-^{Pa}murA(C117R) gent]*	
PAO1/pKH37-5U	PA1140	*[P_{lac}-^{Pa}murA(C117S) gent]*	
PAO1/pKH37-3D	PA1141	*[P_{lac}-^{Pa}murA(I119F) gent]*	
PAO1/pKH37-1B	PA1142	*[P_{lac}-^{Pa}murA(L126P) gent]*	
PAO1/pKH37-1C	PA1143†	*[P_{lac}-^{Pa}murA(V166A) gent]*	
PAO1/pKH37-1E	PA1144	*[P_{lac}-^{Pa}murA(E193G) gent]*	
PAO1/pKH37-2B	PA1145	*[P_{lac}-^{Pa}murA(D234A) gent]*	
PAO1/pKH37-4N	PA1146	*[P_{lac}-^{Pa}murA(I236T) gent]*	
PAO1/pKH37-4L	PA1147	*[P_{lac}-^{Pa}murA(T240I) gent]*	
PAO1/pKH37-5D	PA1148	*[P_{lac}-^{Pa}murA(D308G) gent]*	
PAO1/pKH37-1D	PA1149†	*[P_{lac}-^{Pa}murA(D308N) gent]*	
PAO1/pKH37-5N	PA1150	*[P_{lac}-^{Pa}murA(Q310R) gent]*	
PAO1/pKH37-5L	PA1151	*[P_{lac}-^{Pa}murA(E328G) gent]*	
PAO1/pKH37-2J	PA1152	*[P_{lac}-^{Pa}murA(V330A) gent]*	
PAO1/pKH37-2H	PA1153	*[P_{lac}-^{Pa}murA(F331L) gent]*	
PAO1/pKH37-4J	PA1154	*[P_{lac}-^{Pa}murA(R334C) gent]*	
PAO1/pKH37-1L	PA1155	*[P_{lac}-^{Pa}murA(R334S) gent]*	
PAO1/pKH37-3G	PA1156†	*[P_{lac}-^{Pa}murA(D372G) gent]*	
PAO1/pKH37-3C	PA1157	*[P_{lac}-^{Pa}murA(A375) gent]*	
PAO1/pKH37-4I	PA1158	*[P_{lac}-^{Pa}murA(L379P) gent]*	
PA1162	PA1162	*^{Pa}murA(C117S) attTn7::$P_{TOPLACUV5}$-^{Pa}murA*	
PA1118/pHerd20T	PA1164	*attTn7::$P_{TOPLACUV5}$-^{Pa}murA [P_{ara}-empty amp]*	
PA1162/pHerd20T	PA1167	*^{Pa}murA(C117S) attTn7::$P_{TOPLACUV5}$-^{Pa}murA [P_{ara}-empty amp]*	
PA1118/pKH269	PA1242	*attTn7::$P_{TOPLACUV5}$-^{Pa}murA [P_{ara}-^{Pa}murA(G58D) amp]*	

PA1162/pKH269	PA1243	^{Pa}*murA(C117S) attTn7::*$P_{TOPLACUV5}$-^{Pa}*murA* [P_{ara}-^{Pa}*murA(G58D) amp]*	

[a] Brackets indicate genetic material encoded by a plasmid.

†The indicated strain also contains a silent mutation in the plasmid-encoded *murA* open reading frame. See Table S2 for details.

Supplementary Table 4 - Primers used in this study

Primer	Sequence
15	ACTGTTGGGAAGGGCGATCAAA
67	CACAGCATAACTGGACTGATTTC
68	GCACATCGGCGACGTGCTCTC
76	ACACTTTATGCTTCCGGCTC
K5	AGGAGGAATTCTCGGCTACGATCAATACCG
K6	CGATGGGAGGGGTGTTCTCCAGCCACAGAATCAGATTCTT
K7	AAGAATCTGATTCTGTGGCTGGAGAACACCCCTCCCATCG
K8	AGGAGTCTAGAGCCCAGCTTGAGAACGAAG
K9	AGGAGGAATTCCCGTTCGAAGGTCGCAAG
K10	CATGGCTTTGGCCGCTTAAG
K11	TTAAGCGGCCAAAGCCATGCATCATCACCATCACCACATCAAACAACGCACCTTGAAG
K12	AGGAGTCTAGAGCCGGGCGCATATAGGAAA
K15	AGGAGAAGCTTGCCTCCAAATATGGAAAGGA
K17	TGACCTCCTTGACGAAGGAA
K21	GTCCAGCCGGTCGTAACG
K24	AGGAGGAATTCAGGAGGAATACACCATGCATCATCACCATCACCACATCAAACAAAGGACACTT AAACGTAT
K25	AGGAGAAGCTTTGTCGTTATGCCAGTACAGC
K27	AGGAGGAATTCAGGAGGAATACACCATGCATCATCACCATCACCACATCAAACAACGCACCTTG AAG
K54	AGGAGGGTACCTTAAGCGGCCAAAGCCATG
K55	AGGAGGCATGCGCCTCCAAATATGGAAAGGA
K78	AGGAGAAGCTTCCATCCTGGCCGAGGAACA
K79	GCGAATGCCGTCGACCAGGGCGATCAGGGTCTGGCT
K80	AGCCAGACCCTGATCGCCCTGGTCGACGGCATTCGC
K81	AGGAGGGATCCTTCCTTGAGCTGCCTGGTC
K98	AGGAGCCCGGGCACGGGGGATCATTGCAAT
K100	GACCGGCGATCCGGCAAAT
K106	TTCATGGAGATGAACTGGGC
K108	GATCGCGCTACCGCCCGG
K109	CCGGGCGGTAGCGCGATC
K112	AGGAGGCATGCACAGCCTCCGCTAGCCCG
K148	AGGAGCCCGGGACGGGAGGAAAGATGGACTACAAAGACGACGATGATAAGGATAAACTGATTA TTACCGGCG
K149	AGGAGAAGCTTACAGCCTCCGCTAGCCCG
K166	AGGAGGAATTCTACGACAACCAGGACATCCG
K167	ACCGCCGGTAATAATCAGTT
K168	AACTGATTATTACCGGCGGTAAGATCCGCCGCGTACCGG
K169	AGGAGTCTAGATGGAGACCGTCGAAGCGCT
K170	AGGAGGAATTCGATGCCATCGTGGTCAACGT
K171	TTGCAGTTCCAGGCTCACAG
K172	CTGTGAGCCTGGAACTGCAACCTGAACCCAATCTCTACTGAGGG
K173	AGGAGTCTAGAGGCAGCGAGGCGACCGATAA
K174	AGGAGGGATCCACGGGAGGAAAGAATGGATAAACTGATTATTACCGGCG
K175	AGGAGGGATCCACGGGAGGAAAGATGAGCCTGGAACTGCAAGAG
K176	AGGAGGCATGCCGCACCCTCAGTAGAGATTGG
K177	GGATGACCCCCCAGGGCCTTGAA
K178	GCCCGGGTGACTGCAGGAAG
K179	TTCAAGGCCCTGGGGGGTCATCCATGATCAAACAACGCACCTTGA
K180	CTTCCTGCAGTCACCCGGGCCTACACTGCCGCCGCCGG
K183	GGTATATCTCCTTCTTAAAGTTAAACAA
K184	GAATTCGAGCTCCGTCGACA
K187	AAGCGTCAGCAGCAGGTCTA
K188	GAGGATTTCTCCCGCCATGA

K193	TTGTTTAACTTTAAGAAGGAGATATACCATGCATCATCACCATCACCACGATAAACTGATTATTACCGGCGG
K194	TGTCGACGGAGCTCGAATTCCTAGCCCGGTACGCGGCGG
K202	TTCAAGGCCCTGGGGGGTCATCCATGATCAAACAAAGGACACTTAAA
K203	CTTCCTGCAGTCACCCGGGCTTATGCCAGTACAGCTGAAGG
K204	TTGTTTAACTTTAAGAAGGAGATATACCATGCATCATCACCATCACCACGATAAATTTCGTGTTCAGGGG
K205	TGTCGACGGAGCTCGAATTCTTATTCGCCTTTCACACGCTC
K218	AGGAGGGATCCCATTGACGAACTTGGTGGCC
K223	TTCAAGGCCCTGGGGGGTCATCCGTGAAACAGCGTACTCTCAAT
K224	CTTCCTGCAGTCACCCGGGCTTATGTCACACTCACGTATGGA
K225	TTGTTTAACTTTAAGAAGGAGATATACCATGCATCATCACCATCACCACGATAAATTTTTAATCACGGGCG
K226	TGTCGACGGAGCTCGAATTCTTAACTTACTCGCTTAATTTTGG
K238	CCAAAATTAAGCGAGTAAGTTAAGAATTCGAGCTCCGTCGACA
K239	TGTCGACGGAGCTCGAATTCTTAACTTACTCGCTTAATTTTGG
K284	CGCATTTCCGGCGCGCGCAACTCGGCGCTGCCG
K285	CGGCAGCGCCGAGTTGCGCGCGCCGGAAATGCG
K286	CCTGCCGGGCGGTCGCGCGATCGGTTC
K287	GAACCGATCGCGCGACCGCCCGGCAGG
K288	GGCGGTTGCGCGTTTGGTTCGCGTCCG
K289	CGGACGCGAACCAAACGCGCAACCGCC
K290	GCGTTCCCCACCAACATGCAGGCCC
K291	GGGCCTGCATGTTGGTGGGGAACGC
K292	TTCCCCACCGACATGCGCGCCCAGTTCATCTCC
K293	GGAGATGAACTGGGCGCGCATGTCGGTGGGGAA
K294	GACGGTCTTCGAGAACTGCTTCATGCATGTTTAC
K295	GTAAACATGCATGAAGCAGTTCTCGAAGACCGTC
K296	GACGGTCTTCGAGAACAGCTTCATGCATGTTTAC
K297	GTAAACATGCATGAAGCTGTTCTCGAAGACCGTC
K379	GATCGAACTGTTCGACCGCATGGGCGTGC
K380	GCACGCCCATGCGGTCGAACAGTTCGATC
K381	GTGGCTACGAGTGCATCAAGGAGAAACTCCAGCTGC
K382	GCAGCTGGAGTTTCTCCTTGATGCACTCGTAGCCAC

Supplementary Table 5 – gBlock sequences used in this study

gBlock	Sequence[a]
^{Mku}*lpxC*	TTCAAGGCCCTGGGGGGTCATCCCCCGGATCCAATGCGCCGAGTCTGGCGTCGGAAGCCAG<br>TGCAGTGCGCTCCCGTACCCTGAAAACTTCGATCGGTTGTACGGGGGTGGGACTTCATTCAG<br>GTGCCAAAGTTACGATGGTCCTGCATCCGGCCGAGCCTGGAACGGGGATTCGCTTCCGCCGT<br>GTGGATATTAACGGTGGCGGAGCTATCGTTCCTGCAATCTGGTCGGCTGTACATGATACTCGT<br>ATGAACAGCTGCCTGAAAAACGACGATGGTATCGTAGTAGGCACCGTAGAGCATCTGATGTC<br>GGCGTTGGCTGGGATGCAGATTGATAACTGTTTGATCGACATCAATGGTCCTGAAGTCCCTGT<br>AATGGATGGATCTGCTGCGCCGTTCCTTTTTTTAATTGAGTGCGCAGGCGTGGTCGAACAGTC<br>TGCTCCACGCCAAGCCATTAAAGTTTTAAAGCGCGTCTCAGTGAAGGATGGCGATCGCGTAG<br>CGTCGTTAACCCCAAGCTCTGGCTTTTCGATCCGCTTTGAAATTGACTTTGGGGCATCTGCTA<br>TCAGTCGTCAAGAGTTTTTCGTGAATCTGAGCCGCGGCACTTTCAAAAGCGAAATTAGTCGCG<br>CGCGCACCTTCGGTTTTGAACAAGAGGTGGCTTTTCTGCGTGCAAATGGTCTTGCTCGTGGG<br>GGTAGCTTGGATAACGCAGTCGTCATTGACTCAACGGGAACGCGCGTCCTTAACGATGAGGG<br>CCTGCGTTATACCGACGAGTTCGTGCGTCACAAAGTCCTGGACGCGGTTGGAGATTTATATTT<br>AGCTGGAGCCCCCTTGATTGGTCATTTTCATGGGATTCGTTGCGGCCATGCCTTAAATAATCA<br>GTTACTTCGCGCTTTATTTGCCGATCAGACGGCATGGACACTGACGACCGTGGCCCCAGGTT<br>CTGCCGCGGCACCTTTCGCAGCTGAACCTCAACGTGCCGCTTTAAGTGCG**TAA**GCCCGGGTG<br>ACTGCAGGAAG
^{Xba}*lpxC*	TTCAAGGCCCTGGGGGGTCATCCCTGAAGCAGCGCACATTGAAGAACTCGATCCGCGCGACT<br>GGAGTTGGTCTTCATACTGGGAAGAAGGTCTTGATGGTATTACGTCCCGCCCCAGCAGACAC<br>GGGGATTGTGTTTCAACGTACCGACTTGGACGAGCCTGTGGATATCCCAGCCCGCGCGGGTA<br>ACGTCACCGAGACCACACTGGGCACCACACTGACTGTTGGCGAGGCTCGCGTCTCGACTGTC<br>GAGCACCTTATGAGTGCATTAGCTGGCCTGGGTATTGACAACTTATATGTTGAACTTTCTGCG<br>GGGGAGGTCCCGATTATGGACGGCTCAGCAGGGCCATTCGTCTTCTTACTGCAAAGCGCTGG<br>CATCGAGGAGCAGAATGCGCCGAAACGTTTTGTTCGCATCAAGAAAAGCGTCAAAGTCGAGG<br>ACGGAGATAAATGGGCTCGTTTCGATCCTTACGACGGGTTCAAAGTAAACTTCGAAATTGAGT<br>TCGATCATCCAGTCTTTAAGCGCCGCTCGCAGGTTGCGTCAATGGACTTTAGTACCACTACCT<br>TTTTGCGTGAGGTGAGTCGTGCCCGCACCTTTGGATTTATGCGCGACCTTGAATACATGCGTT<br>CTCGCAATCTGGCATTGGGGGGCAATCTGGATAACGCCATTGTACTTGACGATTACCGTGTAC<br>TGAACGAAGACGGGCTGCGCTACGAAGATGAATTTGTCAAGCACAAAATCTTGGATGCTATTG<br>GTGACTTGTATCTTCTTGGTCATTCGCTGATTGGGGAGTTCTCTGGTTATAAGTCAGGGCATG<br>GTCTTAATAATCGTTTGTTACGTACCTTGGTTGCGGACGCTTCAGCCTGGGAAGAAGTCACTT<br>TTGAAACGCTGCAAGATGCACCAATTAGCTACGTGGCTGCTGCTGCTGCCACCGCG**TAA**GCC<br>CGGGTGACTGCAGGAAG
^{Lpn}*lpxC*	TTCAAGGCCCTGGGGGGTCATCCATCAAGCAGCGTACACCCAAAAAGGTCATCCAGGCGACG<br>GGTGTTGGTTTACATAGCGGGGAAAAGGTTCTTCTTACTCTGCGTCCAGCCCCTGTCAACACT<br>GGCATTGTTTTCCGCCGCGTGGATCTTTCGCCAGTGGTAGAAATCCCAGCATCTTACGAGTAT<br>GTAGGTGATACTATGTTGTGTACCACCCTGCATCACGGAAAAGTTAAAATCGCGACAGTAGAA<br>CACCTGCTGTCTGCACTGGCAGGGTTGGGAATTGACAACGCCTACATTGATGTGAACGCCCC<br>CGAGATTCCGATTATGGACGGTAGTGCGGCACCATTCGTATTTCTGATTCAATCAGCAGGTAT<br>TCGCGAACAGAACGCGGCTAAGCGTTATATTCGCATCTTAAAGCCGATTCGTGTTGAAGAAAA<br>CGGAAAATATGTACAATTTTTACCCCACAAGGGGTATAAGATTACTTTCACCATTGGGTTCGAG<br>CACCCAGTGTTTAATGATCGCCCGCAGACAGTGAGCTTCGACTTTTCCGGTACTTCTTATGTG<br>AAGGAAGTCTGCCGTGCCCGCACCTTTGGTTTCTTGTCTGACTACGAAAAGCTTCGTGAATGT<br>GATTTGGCAAAGGGGGGATCACTTGACAACGCGATTGTCGTAGATGACTACCGCGTATTGAAT<br>GAGGACGGATTACGCTTCGAGTCTGAGTTCGTAACCCATAAAGTACTTGACGCAATCGGGGAT<br>TTGTACTTATTAGGCAGCAGTTTGATCGGGGCGTTCGAGGGATACAAGTCAGGGCACGAGCT<br>GAACAATCGCCTGCTGCGCGAATTGATGGTTCGTCAAGATGCGTGGGAATATACCTACTTCGA<br>TACAGAGAATTACTTACCCGCTGTACATCCCGAATATTATCCTGTGGAAGCC**TAA**GCCCGGGT<br>GACTGCAGGAAG
^{Mku}*murA*	TTGTTTAACTTTAAGAAGGAGATATACCATGCATCATCACCATCACCACGATCGTATTCGTATC<br>ATTGGAGGCACGCCCTTAAAAGGCACAATTACGATCGGTGGCGCTAAGAACGCTGCCCTTGC<br>CTTGATGCCAGCGTGTTTGTTGACTGACGAAACTTTGTCTCTTGCAAACCTTCCCCACCTGGT<br>AGACATCACTACAATGGCGAACCTTCTGGCACAACATGGAGTCGGAATGATTCTGAATGGAGA

	CGCGGCGAATGGCGGACATACCGGCCGCGTGCTTGAGTTGACTGCCGCGGAAATCACGAAT<br>ACTACTGCCCCGTATGATCTGGTGCGTAAGATGCGTGCCTCGGTCCTTGTCTTGGGTCCCCTT<br>TTGGCTCGCTGTGGACAGGCGCGCGTTTCTTTGCCTGGCGGGTGTGCAATCGGAACTCGCCC<br>CGTTGACTTGCACCTGAAAGCGTTGGAACAAATGGGTGCAGTGATCGAGCTGGAGGAAGGCT<br>ACATCGTAGCGCACGTTCGTGGCCGCCTGAAGGGAGCACATATTATTTTCCCCCAGGTAACC<br>GTCGGCGGGACGGAGAACATCTTGATGGCCGCTTCACTGGCTGAGGGGGAAACCGTAATCG<br>CTAACGCCGCTCGTGAGCCAGAGGTCGCGGATTTGGCCCACTGCTTGGTAGCTATGGGGGC<br>TAAAATTGAGGGCATTGGAAGCGGAACCTTGCACATCCAAGGTGTGGACCGCTTGCATGGGG<br>CTCATTACTCGGTTGTGCCTGACCGCATTGAGACTGGGTCCTATGCTGTAGCAGCCGCGATC<br>ACCCGCGGAGACATCGAATTGGTTGGGGCACGCTTTGACCTTATGGAGTCGGTAAACACGAT<br>CTTAACCGAGTGCGGAGTACTGGTGGAGGAAACGCCGCGCGGGATGCGCGTCTGCGCTGAG<br>GGCCGCGATATTGCGGGGGTGGACATTATGACAGAACCCTATCCCGGTTTCCCTACCGATAT<br>GCAGGCTCAGCTTATGGCACTGATGTCCACTTCATCGGGAGCCTCCATGATTACCGAGACAAT<br>TTTTGAAAATCGCTTTATGCACGTTCCTGAAATGACGCGCATGGGAGCGCGTATCAACGTACA<br>TGGCGCTTCAGCTATTGTACGTGGATCGGCGAAATTATCAGGCGCACAAGTAATGGCCACCG<br>ACTTACGCGCGTCCGTTTCGTTAATCTTGGCCGGACTTGCTGCTGAAGGCGAAACAATCGTAA<br>ATCGCGTGTACCACCTTGACCGTGGATACGAACGTGTAGAGGAGAAGTTAGCGGCGTGTGGA<br>GCGCGTATCGAGCGTTTAAAAGCGACGGCTGCCGAG**TAA**GAATTCGAGCTCCGTCGACA
^{Xba}*murA*	TTGTTTAACTTTAAGAAGGAGATATACCATGCATCATCACCATCACCACGATAAACTGATCATT<br>ACAGGTGGTGTGCCTTTAAATGGAGAAATTCGCATCTCAGGTGCTAAGAATGCGGCGCTGCC<br>GATCTTGGCCGCCACATTGCTTAGCGATTCGCCAATGACAGTGGGTAACATTCCACATCTGCA<br>CGACGTTACTACGACTATGGAACTTTTAGGCCGTATGGGTGTGCAACTTGTAGTTGACGAGAC<br>TATGAACATTGAAGTTGATGCAAGTTCCATTCGTGAATTTTACGCCCCATACGAACTGGTCAAA<br>ACAATGCGTGCCTCTATTTTGGTTCTGGGCCCACTGCTTGCTCGCCATGGGGAAGCCTTGGTT<br>TCATTGCCGGGAGGATGCGCCATTGGCTCGCGTCCGGTTAACCTGCACATTCACGGCCTGGC<br>GAAGATGGGTGCTGACATCCATGTTGAGAACGGGTTTATCCGCGCCCGTGCAAAACGTTTAAA<br>AGGCACACGTCTGGTTATGGACTTGGTAACGGTGACGGGCACTGAAAATTTGATGATGGCGG<br>CAACCCTGGCAAAGGGAACAACAGTGATTGAGAACGCTGCTCGTGAGCCTGAGGTTGTTGAT<br>CTTGCGAATTGTTTGATCGCAATGGGGGCTAGCATTGATGGAGCGGGGACCGATACAATCAC<br>CATCGATGGAGTCGACTCGCTTTCTGGTACACACTATAATGTACTTCCGGACCGCATCGAATC<br>TGGCACGTTCCTTGTCGCCGCAGCAATTACTGGTGGTAACGTGAAGATTAAAGACACTCGTCC<br>TGCAATGTTGGAAGCCATCCTGGAGAAACTTGAAGAGGTGGGAGCGGAGTTGGAAATCGGCG<br>ATGATTGGATTCGTTTGAATATGCACGGACGCCGCCCTAAAGCCGTTAGCGTTCGCACAAGTC<br>CGTACCCCGCCTTCCCGACTGACATGCAAGCTCAATTCACGGCACTTAACGCTGTAGCTGAC<br>GGGACTGGTGTAATTCTTGAAACTGTATTTGAGAATCGTTTTATGCATGTACACGAGCTGCAG<br>CGCATGGGTGCCAATATTCGTTTGGAGGGAAACACAGCAGTGACCCAGGGTGTCAAAAAACT<br>GACTGCAGCGCCCGTGATGGCAACTGATCTGCGCGCTTCAGCCTCTCTTGTTTTGGCAGGAC<br>TGGTGGCAGAAGGGGACACAGTAGTAGATCGCATTTACCATATCGATCGCGGTTATGAGGGC<br>ATTGAAGAAAAACTGGCCCGTTTAGGAGCGCGTATCCGTCGTGTTCCCGGA**TAA**GAATTCGA<br>GCTCCGTCGACA
^{Lpn}*murA*	TTGTTTAACTTTAAGAAGGAGATATACCATGCATCATCACCATCACCACGATAAATTACTGATC<br>AATGGTGGTAAAGCTCTTCATGGTGAGGTAGTCATTAGTGGAGCAAAAAATGCGGCTTTACCC<br>ATTATGGCTGCCTCTTTGTTGGCTAGTGACCACGTAACTATTTCGAATGTTCCCCACCTTAAGG<br>ATATTACAACCATGATGGAACTGTTAGGTCAGCTTGGCGCGCACTTAATCGTGGATGAGAAAA<br>TGAATGTCCAGGTAGATTCATCGCAGGTGAACGAGTTTGTGGCCCCCTATGATCTTGTCAAGA<br>CGATGCGTGCGTCAATCTTAGTCTTGGGGCCTATGCTGGCCCGCTTTGGAAAGGCCGACGTA<br>AGCCTGCCCGGTGGGTGCGCCATTGGAACACGCCCCGTCGACCTGCACTTAAAGGCCCTTC<br>GTGCTATGGGCGCGGACATCACAGTCAAGAACGGATACATCAACGCACGTTGTAAAAAGGGC<br>TGCCTGCAAGGCAAACGTTTAATGTTTGATACCGTAACTGTGACGGGAACAGAAAACGTGTTG<br>ATGGCTGCAGTCCTGGCTGAGGGAATTACTACCATCAAGAATGCAGCCCGTGAGCCTGAAGT<br>CGTGGATCTGGCTAACTTCCTTATCCAAATGGGTGCAAAAATTCGCGGCGCAGGTACTTCCAC<br>TATTGAGGTGGAAGGGGTTGAGTCGCTGAACGGAGGGACATACTCCGTCATGTCCGATCGTA<br>TCGAGGCTGGAACATATTTGGCAGCCGGTGCATTGACGCGCGGCCAGGTAACTGTTAAAAAA<br>GTCCGTCCAGACACATTGTTATCGCAACTTTGCAAGTTTGAGGAAGCGGGAGCCGAATTGACA<br>ATCGGGGAAGACTGGGTCAGTCTTAATATGCACAATAAACGCCCGCAGGCTGTAAATATTTCA<br>ACTGCCCCGTATCCTGCCTTCGCTACGGATATGCAAGCCCAATTCATGGCTATGAACAGTGTT

	GCTGAGGGGTCTTCCACTATTATCGAGACTATTTTTGAAAACCGCTTTATGCATGTGCAAGAGT<br>TACAGCGCATGGGGGCCAACATTCAGCTGAATGGAAACACAGCAATTGTTCATGGAGTAGAAA<br>AGTTAACTGGCGCTCCTGTTATGGCTACTGACCTTCGTGCCTCTGCCAGTCTGATTTTGGCTG<br>GATTAGTCGCAGAAGGTGAGACAGTTGTCGAACGTATCTACCACGTAGATCGTGGGTACGAA<br>CGCATCGAAGAGAAATTATCTCTTCTTGGTGCCGATATTAAGCGCGTGAGTGACCGT**TAA**GAA<br>TTCGAGCTCCGTCGACA

[a] The first codon of the open reading frame is underlined and the stop codon is indicated in bold.

SUPPLEMENTARY REFERENCES:

1 Miller, J. Experiments in molecular genetics. *Cold Spring Harbor Laboratory, Cold Spring Harbor, N.Y.* (1972).

2 Meeske, A. J. *et al.* SEDS proteins are a widespread family of bacterial cell wall polymerases. *Nature* **537**, 634-638, doi:10.1038/nature19331 (2016).

3 Rietsch, A., Vallet-Gely, I., Dove, S. L. & Mekalanos, J. J. ExsE, a secreted regulator of type III secretion genes in Pseudomonas aeruginosa. *Proc Natl Acad Sci U S A* **102**, 8006-8011, doi:10.1073/pnas.0503005102 (2005).

4 Hoang, T. T., Karakhoff-Schweizer, R. R., Kutchma, A. J. & Schweizer, H. P. A broad-host-range Flp-*FRT* recombination system for site-specific excision of chromosomally-located DNA sequences: application for isolation of unmarked *Pseudomonas aeruginosa* mutants. *Gene* **212**, 77-86 (1998).

5 Qiu, D., Damron, F. H., Mima, T., Schweizer, H. P. & Yu, H. D. PBAD-based shuttle vectors for functional analysis of toxic and highly regulated genes in Pseudomonas and Burkholderia spp. and other bacteria. *Appl Environ Microbiol* **74**, 7422-7426, doi:10.1128/AEM.01369-08 (2008).

6 Marmont, L. S. & Bernhardt, T. G. A conserved subcomplex within the bacterial cytokinetic ring activates cell wall synthesis by the FtsW-FtsI synthase. *Proc Natl Acad Sci U S A* **117**, 23879-23885, doi:10.1073/pnas.2004598117 (2020).

7 Fivenson, E. M. & Bernhardt, T. G. An Essential Membrane Protein Modulates the Proteolysis of LpxC to Control Lipopolysaccharide Synthesis in Escherichia coli. *mBio* **11**, e00939-00920, doi:10.1128/mBio.00939-20 (2020).

8 Vvedenskaya, I. O. *et al.* Growth phase-dependent control of transcription start site selection and gene expression by nanoRNAs. *Genes Dev* **26**, 1498-1507, doi:10.1101/gad.192732.112 (2012).

9 Choi, K. H. *et al.* A Tn7-based broad-range bacterial cloning and expression system. *Nat Methods* **2**, 443-448, doi:10.1038/nmeth765 (2005).

10 Greener, A. & CaUahan, M. XL1-RED: a highly efficient random mutagenesis strain. *Strategies* **7**, 32-34 (1994).
