# Supplementary material for: Coordination of bacterial cell wall and outer membrane biosynthesis
Source: Nature. 2023 Mar 1;615(7951):300–4. doi: 10.1038/s41586-023-05750-0 (PMC9995270; doi:10.1038/s41586-023-05750-0)
Supplement: Supplementary file 2 — Reporting Summary [file 41586_2023_5750_MOESM2_ESM.pdf]

nature portfolio

Corresponding author(s): Thomas G. Bernhardt

Last updated by author(s): 12/22/2022

Reporting Summary

Nature Portfolio wishes to improve the reproducibility of the work that we publish. This form provides structure for consistency and transparency in reporting. For further information on Nature Portfolio policies, see our Editorial Policies and the Editorial Policy Checklist.

Statistics

For all statistical analyses, confirm that the following items are present in the figure legend, table legend, main text, or Methods section.

n/a	Confirmed	
☐	☒	The exact sample size (*n*) for each experimental group/condition, given as a discrete number and unit of measurement
☐	☒	A statement on whether measurements were taken from distinct samples or whether the same sample was measured repeatedly
☐	☒	The statistical test(s) used AND whether they are one- or two-sided<br>*Only common tests should be described solely by name; describe more complex techniques in the Methods section.*
☒	☐	A description of all covariates tested
☐	☒	A description of any assumptions or corrections, such as tests of normality and adjustment for multiple comparisons
☐	☒	A full description of the statistical parameters including central tendency (e.g. means) or other basic estimates (e.g. regression coefficient) AND variation (e.g. standard deviation) or associated estimates of uncertainty (e.g. confidence intervals)
☐	☒	For null hypothesis testing, the test statistic (e.g. *F*, *t*, *r*) with confidence intervals, effect sizes, degrees of freedom and *P* value noted<br>*Give P values as exact values whenever suitable.*
☒	☐	For Bayesian analysis, information on the choice of priors and Markov chain Monte Carlo settings
☒	☐	For hierarchical and complex designs, identification of the appropriate level for tests and full reporting of outcomes
☒	☐	Estimates of effect sizes (e.g. Cohen's *d*, Pearson's *r*), indicating how they were calculated

Our web collection on statistics for biologists contains articles on many of the points above.

Software and code

Policy information about availability of computer code

Data collection	Covariation analysis was performed using open-source softwares Jackhmmer v3.1b2, EVcouplings v0.0.5, Python v3.8.8, FastTree v2.1.11 and AlphaFold2.
Data analysis	Statistical analyses were performed by Prism 9 (GraphPad Software, LLC.) LC/MS data were analyzed using Agilent MassHunter Workstation Qualitative Analysis software version B.06.00. Protein sequences from pulldown experiments were determined using Sequest ver 28 (rev 13). Structural data were visualized using ChimeraX version 1.1.1 and phylogenetic data were visualized using the interactive tree of life version 5. Microscopy images were processed using ImageJ2 version 2.3.0/1.53q and the MicrobeJ version 5.13l plugin. The distribution LpxC/MurA interaction scores was analyzed using sklearn v1.0.2 and ETE3 v1.3.2.

For manuscripts utilizing custom algorithms or software that are central to the research but not yet described in published literature, software must be made available to editors and reviewers. We strongly encourage code deposition in a community repository (e.g. GitHub). See the Nature Portfolio guidelines for submitting code & software for further information.

Data

Policy information about availability of data

All manuscripts must include a data availability statement. This statement should provide the following information, where applicable:

- Accession codes, unique identifiers, or web links for publicly available datasets
- A description of any restrictions on data availability
- For clinical datasets or third party data, please ensure that the statement adheres to our policy

Data used to generate graphs presented in this work are available as source data. LC-MS/MS source data, microscopy images, and computational intermediates used to derive LpxC-MurA interaction scores can be accessed using the DOI 10.5281/zenodo.7455522. Uniprot accession codes for genes used to generate LpxC-MurA interaction scores can be found in the methods and Source Data 3. All bacterial strains and plasmids developed in this study are available upon request. All code generated in this study can be accessed https://github.com/samberry19/evcomplex-interaction-scoring or by using DOI 10.5281/zenodo.7471436.

Human research participants

Policy information about studies involving human research participants and Sex and Gender in Research.

Reporting on sex and gender	N/A
Population characteristics	N/A
Recruitment	N/A
Ethics oversight	N/A

Note that full information on the approval of the study protocol must also be provided in the manuscript.

Field-specific reporting

Please select the one below that is the best fit for your research. If you are not sure, read the appropriate sections before making your selection.

☒ Life sciences ☐ Behavioural & social sciences ☐ Ecological, evolutionary & environmental sciences

For a reference copy of the document with all sections, see nature.com/documents/nr-reporting-summary-flat.pdf

Life sciences study design

All studies must disclose on these points even when the disclosure is negative.

Sample size	No statistical methods were used to predetermine sample size. In the case of microscopy experiments, all experiments were performed using greater than 100 cells per population spanning multiple fields of view, which is standard for the field. All other experiments reported bulk measurements of population wide behaviors, involving greater than 1x10^8 cells (for in vivo assays) or greater than 1 x 10^12 protein copies (for in vitro assays) as is standard for the field and each experiment was performed at least twice.
Data exclusions	No data were excluded from this study
Replication	All data are from a minimum of two independent experiments. All replication attempts were successful.
Randomization	Samples were not randomized in this study. Covariates were controlled by reproducing the experiments on separate days.
Blinding	We did not blind samples as, after each experimental setup, all measurements and analyses were performed identically across all conditions.

Reporting for specific materials, systems and methods

We require information from authors about some types of materials, experimental systems and methods used in many studies. Here, indicate whether each material, system or method listed is relevant to your study. If you are not sure if a list item applies to your research, read the appropriate section before selecting a response.

Materials & experimental systems

n/a	Involved in the study
☐	☒ Antibodies
☒	☐ Eukaryotic cell lines
☒	☐ Palaeontology and archaeology
☒	☐ Animals and other organisms
☒	☐ Clinical data
☒	☐ Dual use research of concern

Methods

n/a	Involved in the study
☒	☐ ChIP-seq
☒	☐ Flow cytometry
☒	☐ MRI-based neuroimaging

Antibodies

Antibodies used

Anti-His (GenScript A00186-100); Anti-FLAG (Sigma F7425); Anti-RpoA (BioLegend 663104); Goat-anti-Mouse (Rockland 610-1302); Rabbit TrueBlot®: Anti-Rabbit IgG HRP (Rockland 18-8816-33)

Validation

Anti-His and Anti-FLAG antibodies have been used numerous times to detect His and FLAG-tagged proteins by western blot and validation information can be found at the manufacturers' websites (https://www.genscript.com/antibody/A00186-THE_His_Tag_Antibody_mAb_Mouse.html and https://www.sigmaaldrich.com/US/en/product/sigma/f7425, respectively). In addition, we verified that neither antibody reacts with Pseudomonas aeruginosa lysates derived from strains that do not encode a His- or FLAG-tagged protein (see Extended Data 4A). Monoclonal Anti-RpoA Clone 4RA2 has been used extensively in the field to detect the RNA polymerase subunit alpha in diverse gram-negative bacteria and validation can be found at the manufacturer's website (https://www.biolegend.com/it-it/products/purified-anti-e-coli-rna-polymerase-alpha-antibody-14680). Further, this antibody has been shown to react with specifically with purified P. aeruginosa PAO1 RNA polymerase (Ceyssens, P. J. et al. The Phage-Encoded N-Acetyltransferase Rac Mediates Inactivation of Pseudomonas aeruginosa Transcription by Cleavage of the RNA Polymerase Alpha Subunit. Viruses 12, doi:10.3390/v12090976 (2020).)
