# Supplementary material for: Coordination of bacterial cell wall and outer membrane biosynthesis
Source: Nature. 2023 Mar 1;615(7951):300–4. doi: 10.1038/s41586-023-05750-0 (PMC9995270; doi:10.1038/s41586-023-05750-0)

Figure 1b left pannel

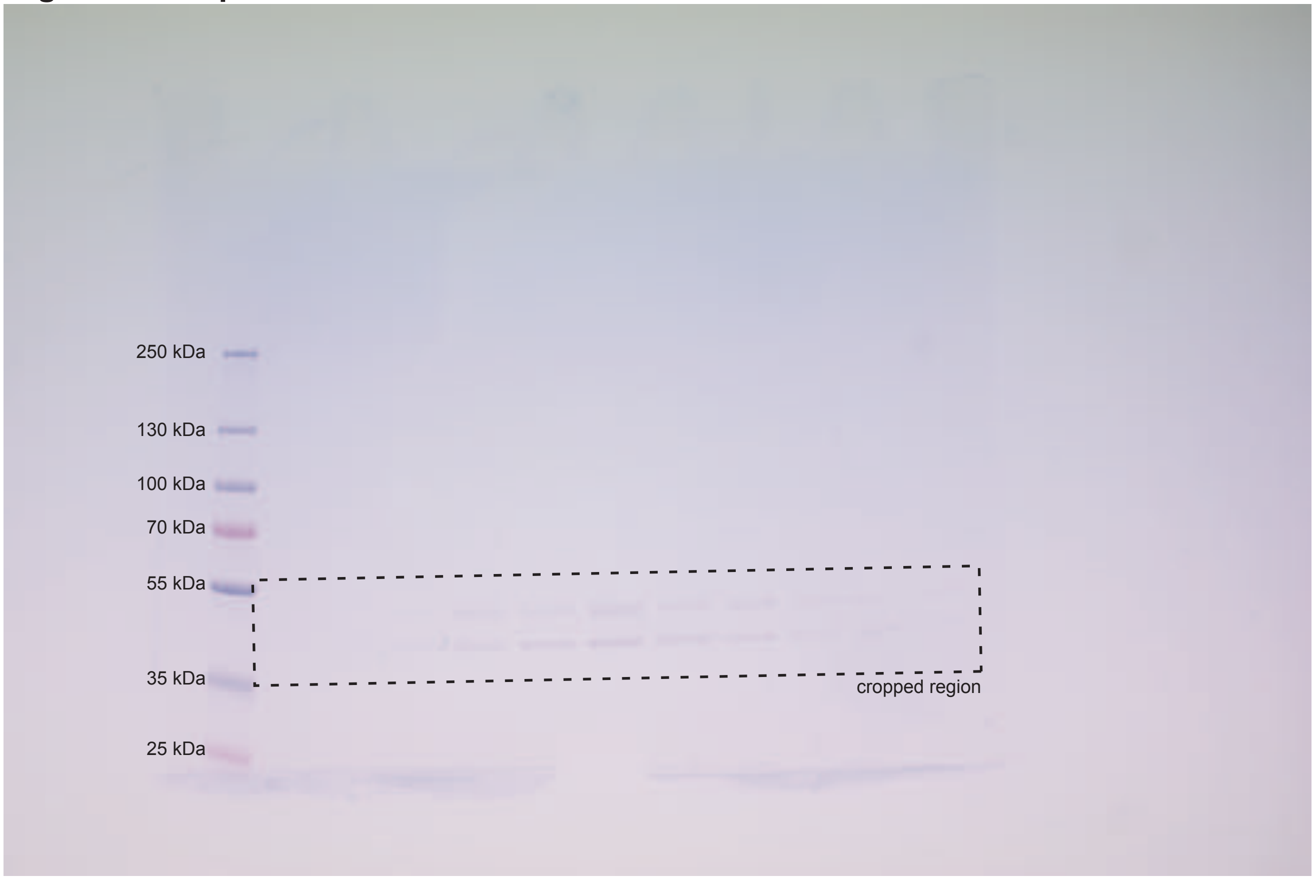

Figure 1b right pannel

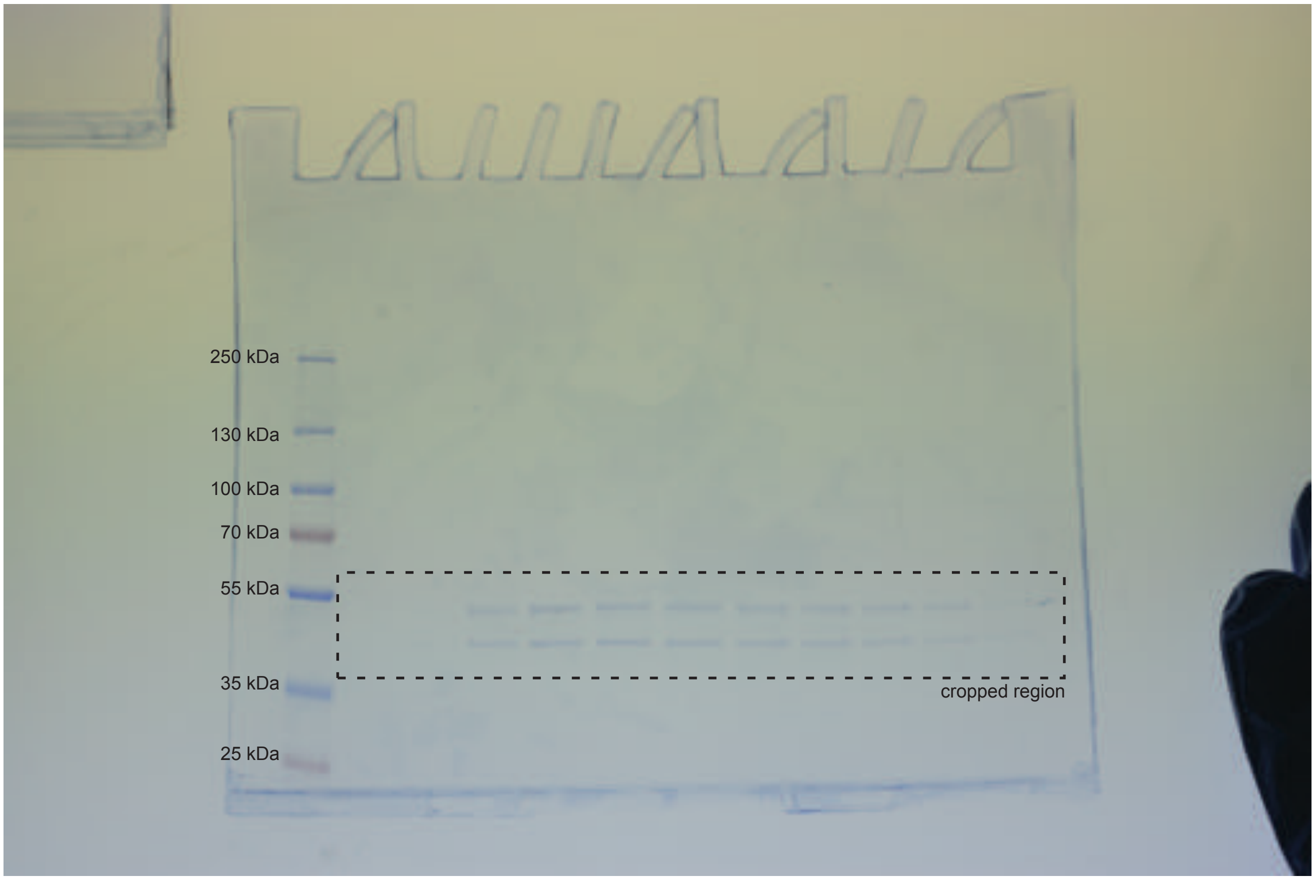

Figure 3b - Mku panel

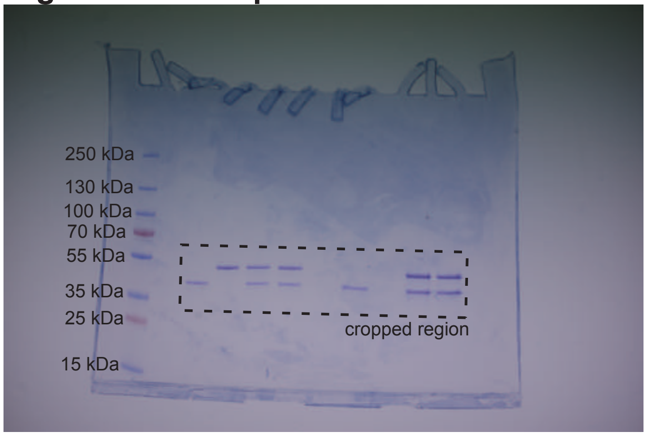

Figure 3b - Xba panel

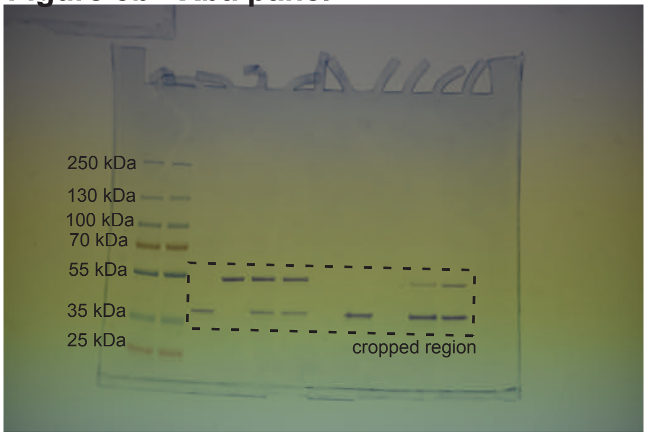

Figure 3b - Lpn panel

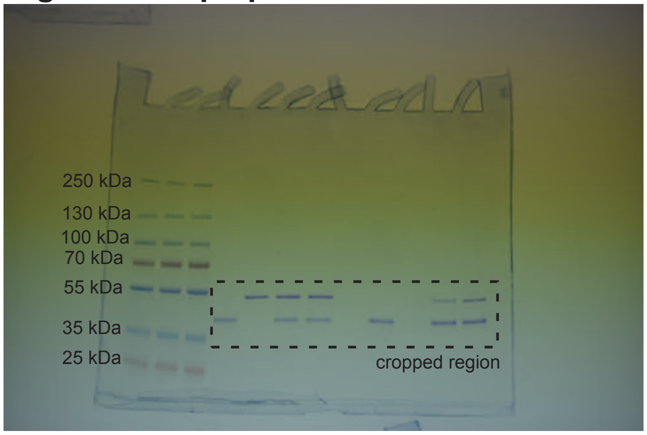

Figure 3b - Aba panel

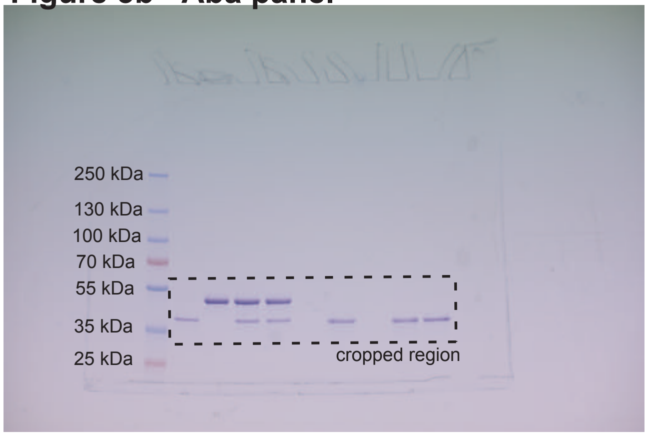

Figure 3b - Eco panel

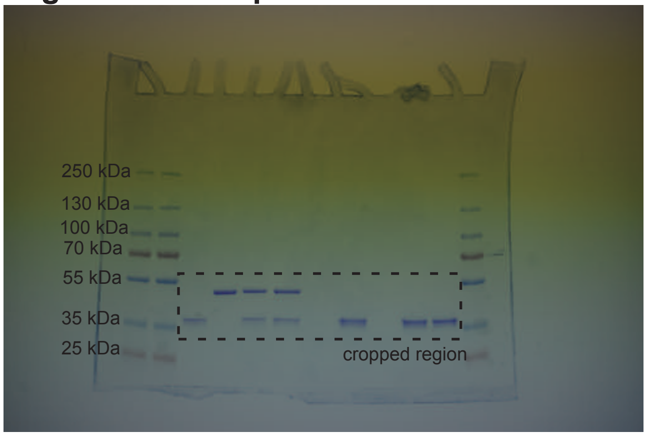

Extended Data 1A - αHis panel

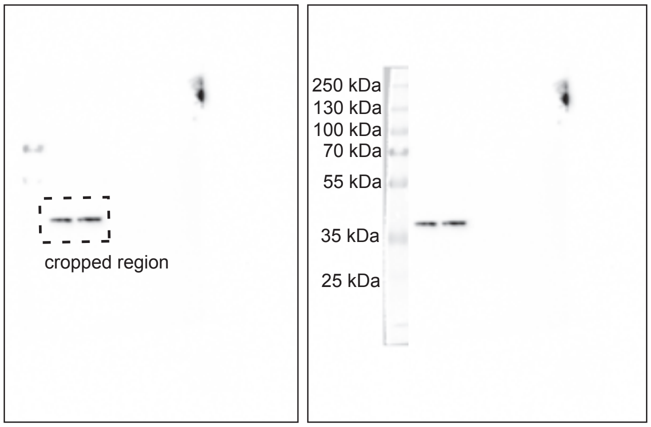

Extended Data 1A - αRpoA panel

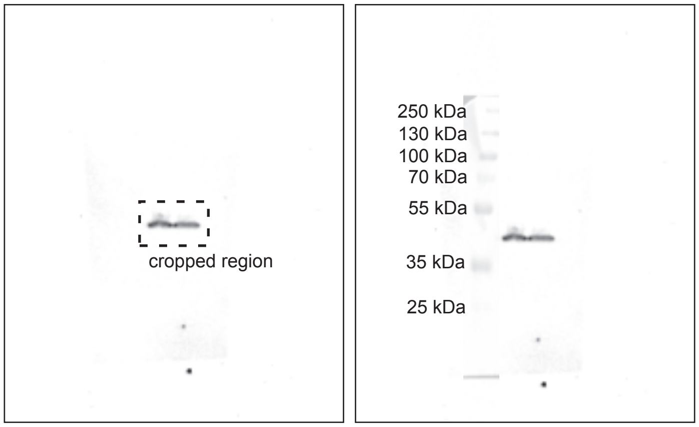

Extended Data 1C - αHis panel

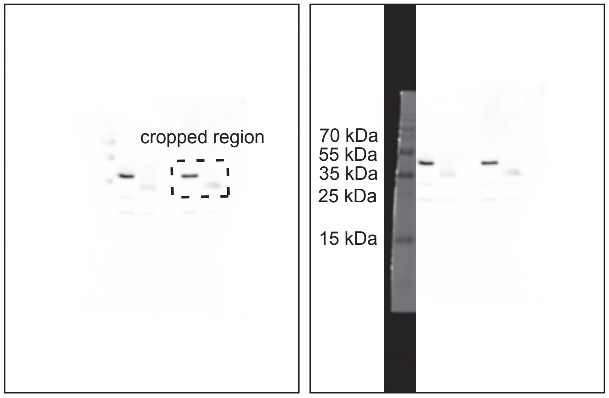

Extended Data 1C - αRpoA panel

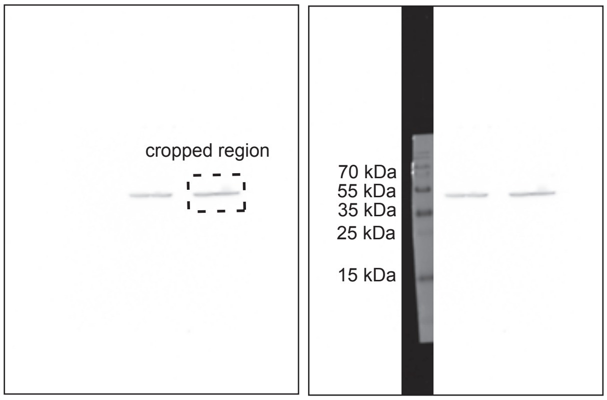

Extended Data 2 - LPS panels

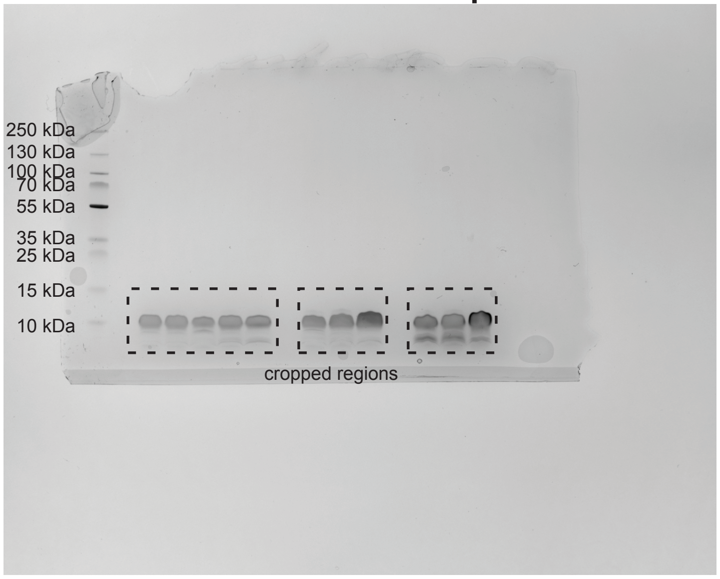

Extended Data 2 - αRpoA panels

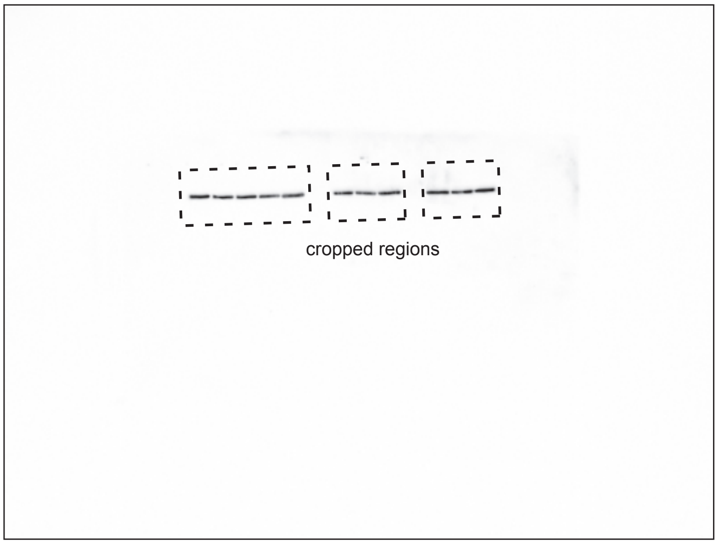

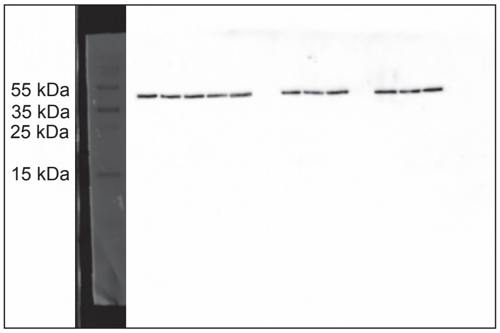

Extended Data 4A - His-LpxC Input

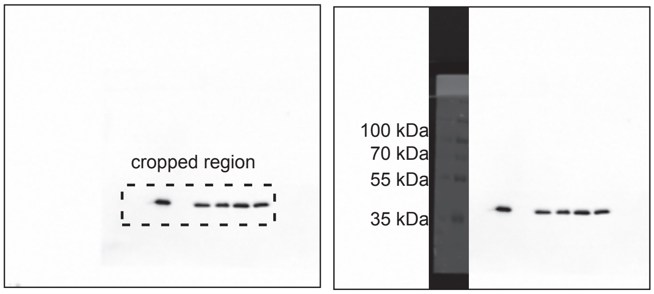

Extended Data 4A - His-LpxC Elution

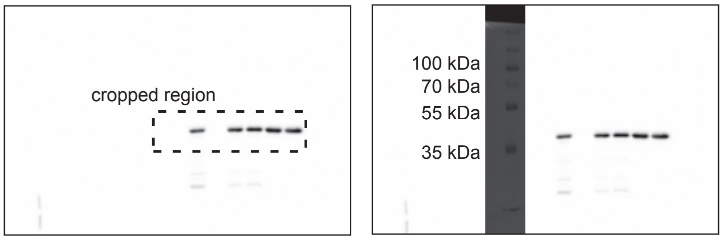

Extended Data 4A - FLAG-MurA Input

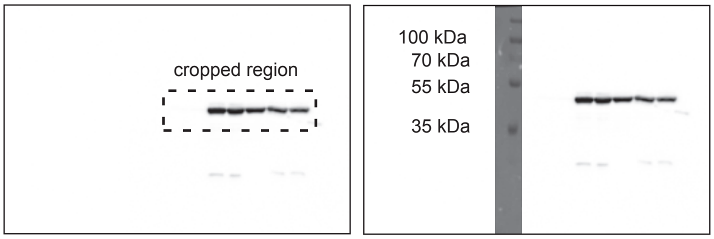

Extended Data 4A - FLAG-MurA Elution

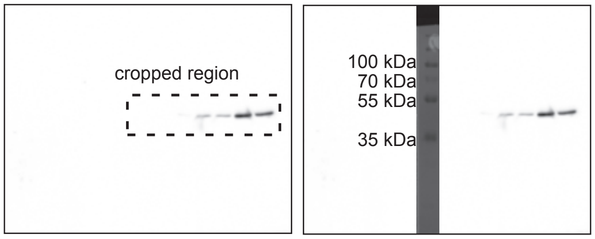

Extended Data 4B - Input

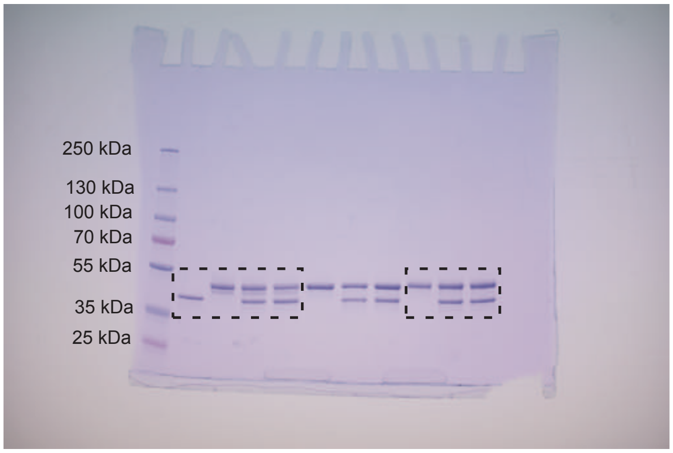

Extended Data 4B - Elution

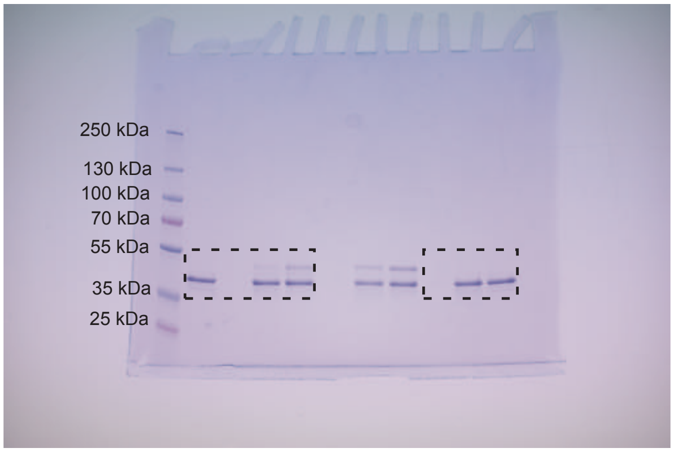

Extended Data 5A

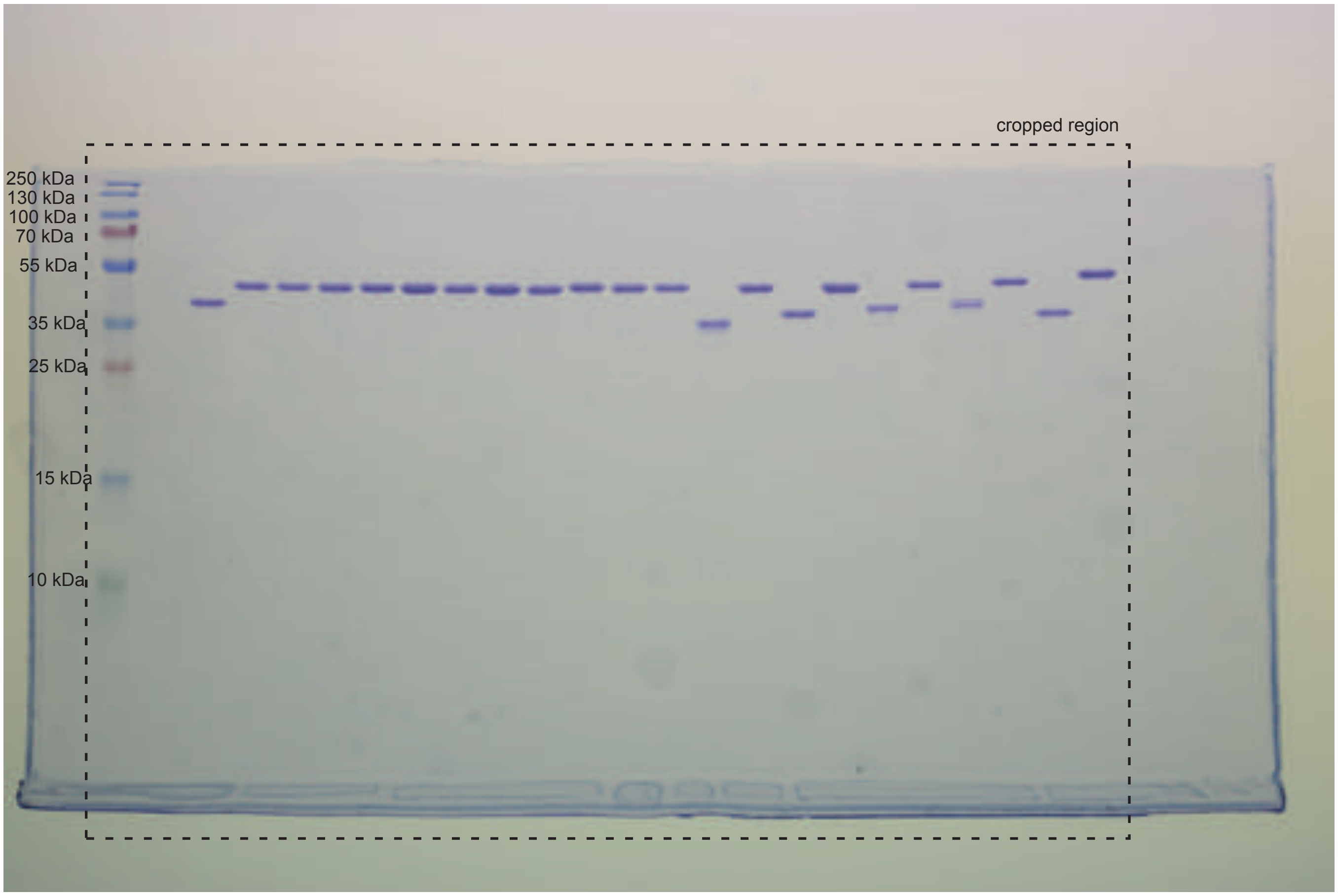

Extended Data 8A - αRpoA panel

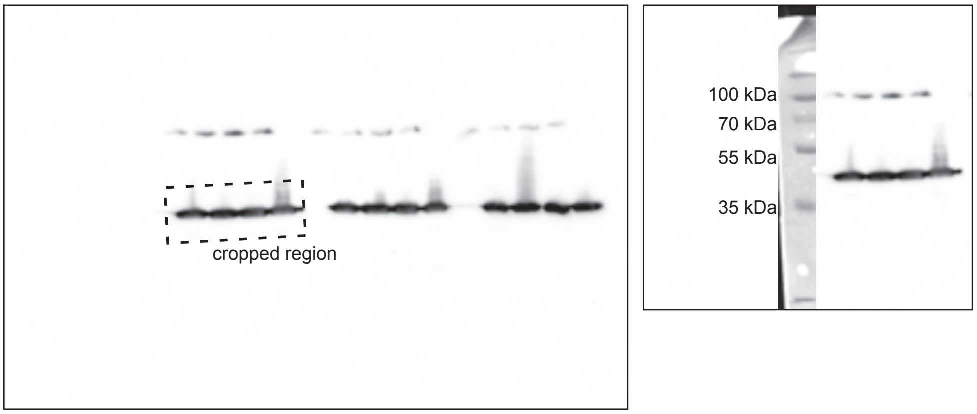

Extended Data 8A - αFLAG panel

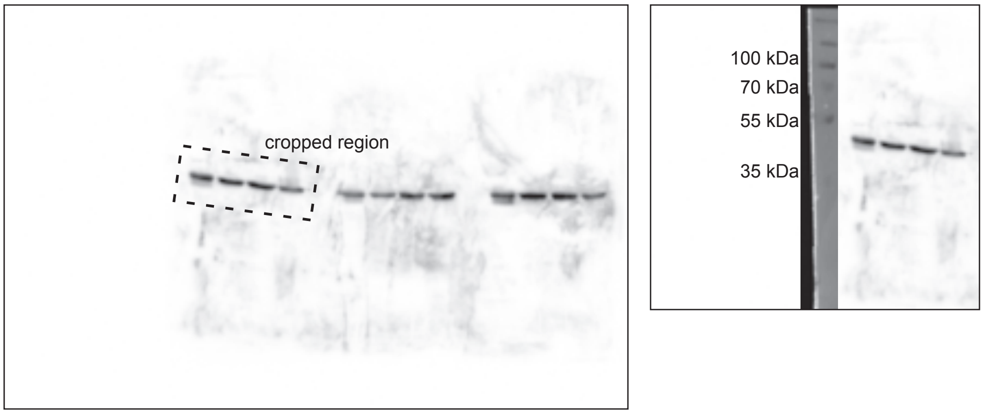

Extended Data 8C - Input panel

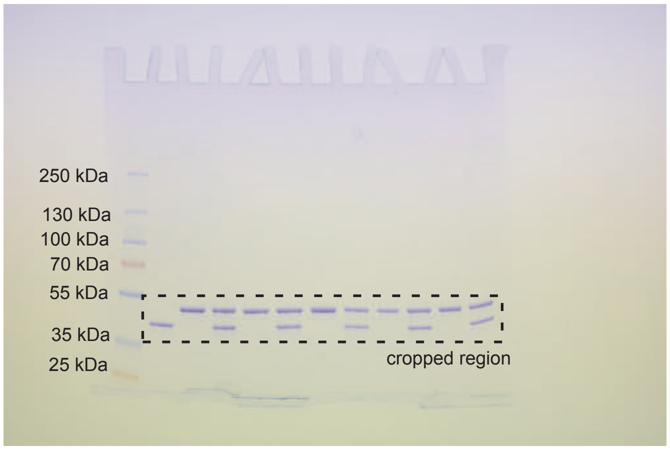

Extended Data 8C - Elution panel

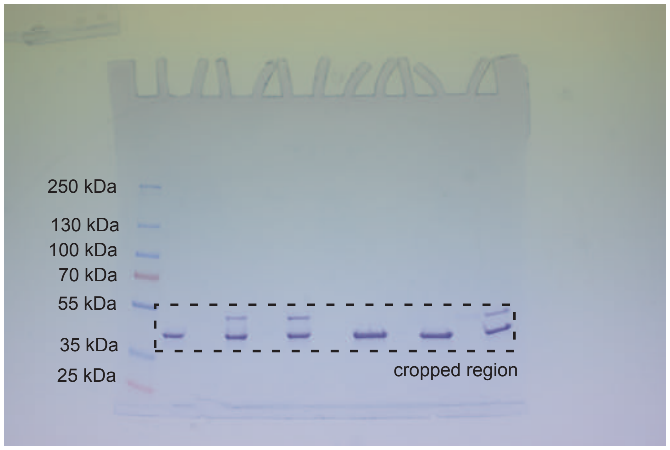

Supplement: Supplementary file 4 — Original source images for all electrophoresis gels reported in this study. [file 41586_2023_5750_MOESM4_ESM.pdf]
